# Feasibility and accuracy of the fully automated three-dimensional echocardiography right ventricular quantification software in children: validation against cardiac magnetic resonance

**DOI:** 10.1007/s00247-025-06330-2

**Published:** 2025-07-18

**Authors:** Qian Liu, Zhiwei Zheng, Yiwei Zhang, Anjun Wu, Jie Lou, Xin Chen, Yating Yuan, Mingxing Xie, Li Zhang, Peng Sun, Wei Sun, Qing Lv

**Affiliations:** 1 Department of Ultrasound Medicine, Union Hospital, Tongji Medical College, Huazhong University of Science and Technology, Clinical Research Center for Medical Imaging in Hubei Province, Hubei Province Key Laboratory of Molecular Imaging, 1277 Jiefang Avenue, Wuhan, 430022 China; 2 Department of Emergency Medicine, Union Hospital, Tongji Medical College, Huazhong University of Science and Technology, 1277 Jiefang Avenue, Wuhan, 430022 China; 3 Department of Ultrasound, Wuhan No.1 Hospital, Wuhan, China; 4 Clinical Research Center for Medical Imaging in Hubei Province, Wuhan, China; 5 Hubei Province Key Laboratory of Molecular Imaging, Wuhan, China; 6 Department of Radiology, Union Hospital, Tongji Medical College, Huazhong University of Science and Technology, Wuhan, China

**Keywords:** Artificial intelligence, Cardiac magnetic resonance, Children, Right ventricular function, Right ventricular volume, Three-dimensional echocardiography

## Abstract

**Background:**

Previous studies have confirmed that fully automated three-dimensional echocardiography (3DE) right ventricular (RV) quantification software can accurately assess adult RV function. However, data on its accuracy in children are scarce.

**Objective:**

This study aimed to test the accuracy of the software in children using cardiac magnetic resonance (MR) as the gold standard.

**Materials and methods:**

This study prospectively enrolled 82 children who underwent both echocardiography and cardiac MR within 24 h. The RV end-diastolic volume (EDV), end-systolic volume (ESV), and ejection fraction (EF) were obtained using the novel 3DE-RV quantification software and compared with cardiac MR values across different groups.

**Results:**

The novel 3DE-RV quantification software was feasible in all 82 children (100%). Fully automated analysis was achieved in 35% patients with an analysis time of 8 ± 2 s and 100% reproducibility. Manual editing was necessary in the remaining 65% patients. The 3DE-derived RV volumes and EF correlated well with cardiac MR measurements (RVEDV, *r*=0.93; RVESV, *r*=0.90; RVEF, *r*=0.82; all *P *<0.001). Although the automated approach slightly underestimated RV volumes and overestimated RVEF compared with cardiac MR in the entire cohort, the bias was smaller in children with RVEF ≥ 45%, normal RV size, and good 3DE image quality.

**Conclusion:**

Fully automated 3DE-RV quantification software provided accurate and completely reproducible results in 35% children without any adjustment. The RV volumes and EF measured using the automated 3DE method correlated well with those from cardiac MR, especially in children with RVEF ≥ 45%, normal RV size, and good 3DE image quality. Therefore, the novel automated 3DE method may achieve rapid and accurate assessment of RV function in children with normal heart anatomy.

**Graphical Abstract:**

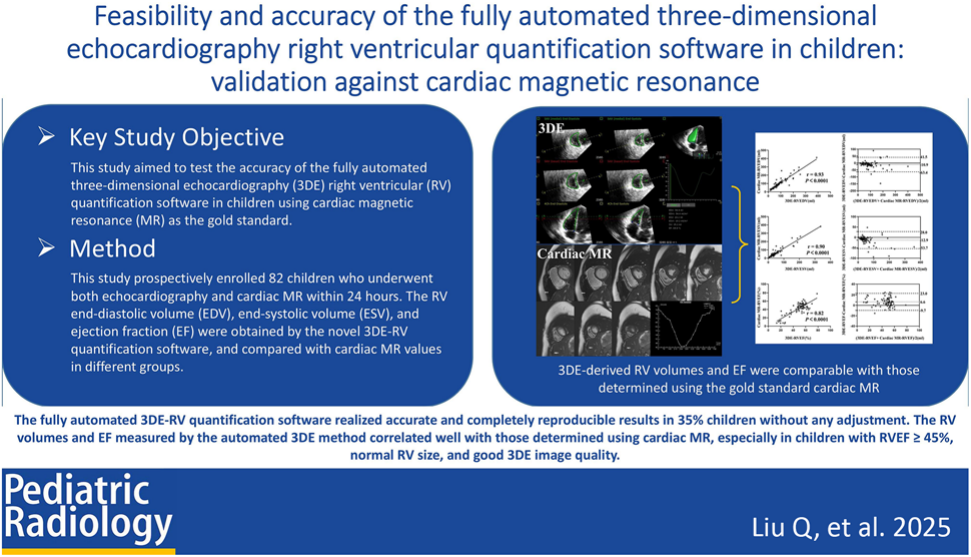

**Supplementary Information:**

The online version contains supplementary material available at 10.1007/s00247-025-06330-2.

## Introduction

Rapid and accurate measurement of right ventricular (RV) function is vital for the diagnosis, prognostic assessment, and clinical management of various cardiopulmonary diseases [1–7]. Accurate assessment of RV volume and function remains challenging due to its complex crescent shape. Cardiac magnetic resonance (MR) imaging is the gold standard for assessing RV volumes and ejection fraction (EF). Nevertheless, owing to high costs, prolonged scanning times, and numerous contraindications [8], cardiac MR imaging is unsuitable for all patients, especially for children who cannot cooperate with long-term examinations and periodic breath-holding.

Echocardiography is a convenient and widely used imaging technique for assessing RV function in clinical practice. The accuracy of three-dimensional echocardiography (3DE) for quantification of RV volume and function compared to the gold standard cardiac MR has been demonstrated in numerous studies [9–11]. However, the currently used semi-automated 3DE-RV quantification software requires a tedious manual operation process and specialized training, which is time-consuming, and limits its popularity in clinical routine. A fully automated 3DE-RV quantification software tool developed in recent years enables circumvention of these limitations. The fully automated 3DE-RV quantification software is an artificial intelligence software based on a machine learning algorithm. It has been shown to achieve fully automatic RV 3DE image processing and function quantization in adult cohorts [6, 12, 13]. However, it remained unclear whether this fully automated 3DE-RV quantification software could accurately assess the RV volumes and function in children.

The group of children included in this study exhibited significant variability in terms of developmental stage and body shape. Furthermore, children with cardiopulmonary disease are characterized by relatively fast heart rates and poor respiratory coordination [14]. These factors pose substantial challenges for fully automated 3DE software. Given these unique characteristics, there is an urgent need to investigate whether this software can accurately quantify RV function in children to meet the growing clinical needs. Therefore, this study aimed to test the feasibility and accuracy of fully automated 3DE in assessing RV volumes and function in children using cardiac MR as the gold standard.

## Material and methods

### Population and study protocol

This study prospectively enrolled 90 consecutive children (aged <18 years old) who were referred for both echocardiography and cardiac MR imaging within 24 h. Children with clinical instability, arrhythmia, or heart-related treatment administered between the cardiac MR and echocardiography examinations were excluded. RV volumes and EF were measured in all recruited children using the fully automated 3DE software and cardiac MR, respectively. The study was approved by the Ethics Committee of Tongji Medical College, Huazhong University of Science and Technology.

All children were divided into subgroups according to the following criteria: (1) Based on cardiac MR-derived RVEF, two subgroups with RVEF ≥ 45% and RVEF <45% were classified [15]; (2) two additional subgroups with RV dilatation (age <7 years old, right ventricular end-diastolic volume index (RVEDVi) ≥ 91 ml/m^2^ in boys or ≥ 89 ml/m^2^ in girls; age 7 ~ 18 years old, RVEDVi ≥ 114 ml/m^2^ in boys or ≥ 85 ml/m^2^ in girls) and normal RV size (age <7 years old, RVEDVi <91 ml/m^2^ in boys or <89 ml/m^2^ in girls; age 7 ~ 18 years old, RVEDVi <114 ml/m^2^ in boys or <85 ml/m^2^ in girls) based on their RVEDVi [16]; (3) finally, children were categorized into three subgroups by 3DE image quality: good, fair, and poor (as outlined below).

### Three-dimensional echocardiography imaging

**Fig. 1:**
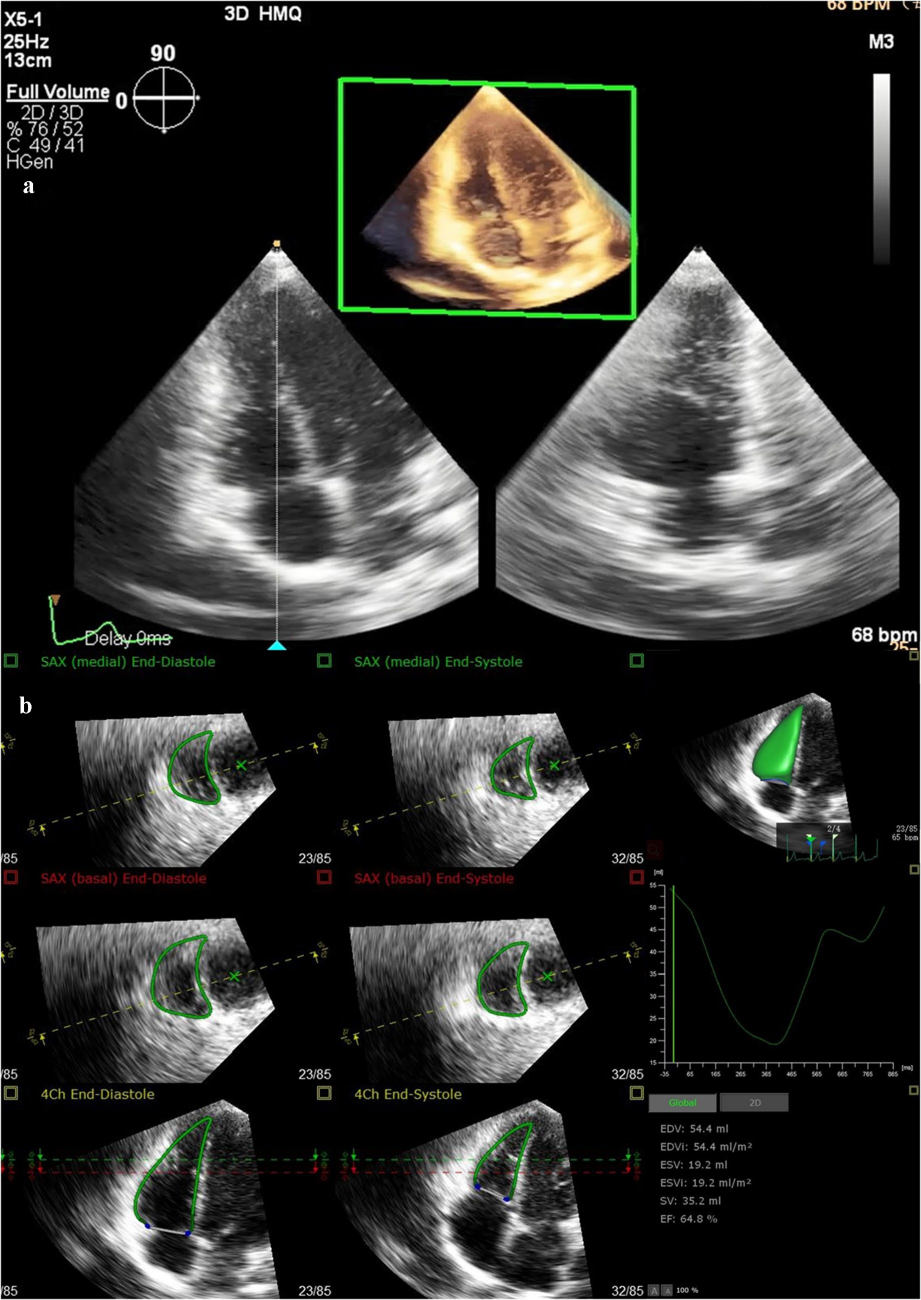
Three-dimensional echocardiography (3DE) depicting the right ventricle in a 6-year-old boy with noncompaction cardiomyopathy. **a** The 3DE images were obtained on the RV-focused apical four-chamber view in full-volume mode. **b** Starting the software, the software automatically tracked the right ventricular (RV) endocardial border (*green line*) at the end-diastole and end-systole, and generated a 3DE-RV cast, RV volume curve, and volumetric data

Echocardiographic imaging was performed from the apical transducer position using the EPIQ 7 C system (Philips Healthcare, Andover, MA), equipped with an X5-1 phased-array transducer. In principle, all children were positioned in the left lateral position with simultaneous electrocardiogram (ECG) recording. For young children unable to maintain the body position, image acquisition was conducted in the supine position. The 3-D echocardiographic acquisitions were obtained using the RV-focused apical four-chamber view [17] in full-volume mode at a median frame rate of 25 frames/s, gathered over four cardiac cycles during a breath-hold period (Fig. 1). For children unable to cooperate with breath-holding, normal breathing was maintained, and acquisitions were performed during stable ECG status. The entire RV cavity was encompassed within the scanning volume through the cardiac cycle. Imaging depth and sector width were optimized to maximize frame rate. All the 3-D echocardiographic images adhered to established guidelines [17, 18]. The 3DE datasets were digitally stored for offline analysis.

### Three-dimensional echocardiography imaging analysis

The RV-focused 3DE full-volume datasets of all children were analyzed using the new fully automated RV quantification software (3-D Auto RV, Qlab 15, Philips Healthcare) by an experienced investigator blinded to the measurements of cardiac MR. After initiating the program, the software automatically performed a view adjustment and constructed a 3-D endocardial cast of the right ventricle at the end-diastole using the Heart Model segmentation algorithm. Subsequently, the software automatically performed 3-D speckle tracking analysis on the RV endocardial border and generated a 3-D dynamic surface and a volume curve of RV (Fig. 1), from which the RV end-diastolic volume (RVEDV), RV end-systolic volume (RVESV), and RVEF were automatically determined.

**Fig. 2:**
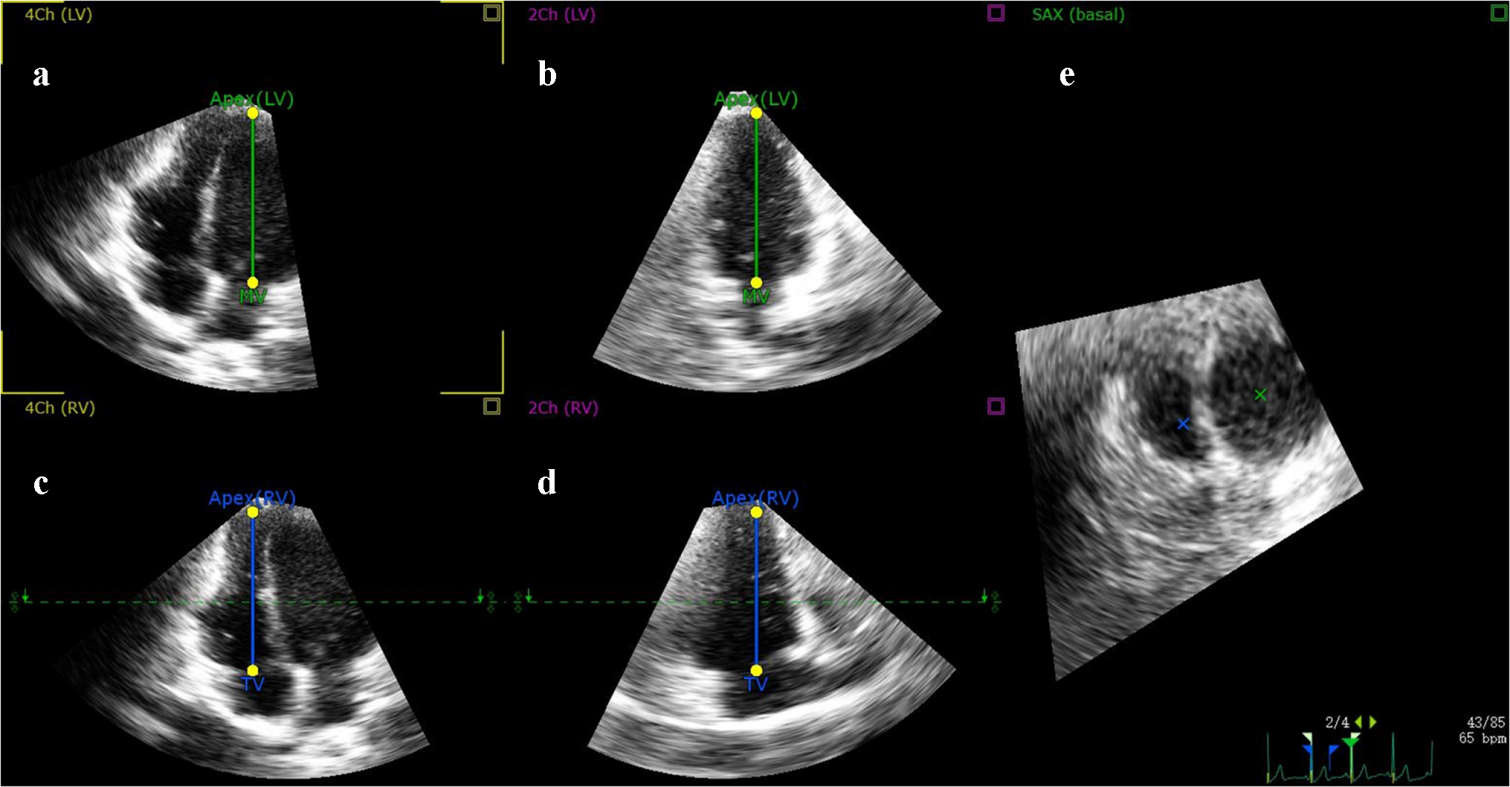
The software enables automatic generation of multiplanar reformations from three-dimensional echocardiography (3DE), depicting the right ventricle of a 6-year-old boy diagnosed with noncompaction cardiomyopathy. When necessary, the operator edited the initial landmarks—including the left ventricular and right ventricular (RV) apex locations, along with the mitral and tricuspid annulus hinge points—using the following standard views: **a** apical four-chamber; **b** left ventricular two-chamber; **c** RV-focused apical four-chamber; **d** RV inflow-outflow; and **e** RV short-axis

Importantly, after the fully automated analysis was completed, the accuracy of the boundary detection was reviewed by carefully checking the RV contours throughout the cardiac cycle. When the automated analysis was judged to be accurate, no manual editing was required. In contrast, when the automated analysis was judged to be inaccurate, the RV endocardial tracking process required manual adjustment. To optimize the fully automated analysis results, the initial landmarks could be edited by the operator, which included the location of the left ventricular and RV apex, the mitral and the tricuspid annulus hinge points (Fig. 2), and further adjustments could be made to the RV endocardial borders (Fig. 1).

### Image quality assessment

**Fig. 3:**
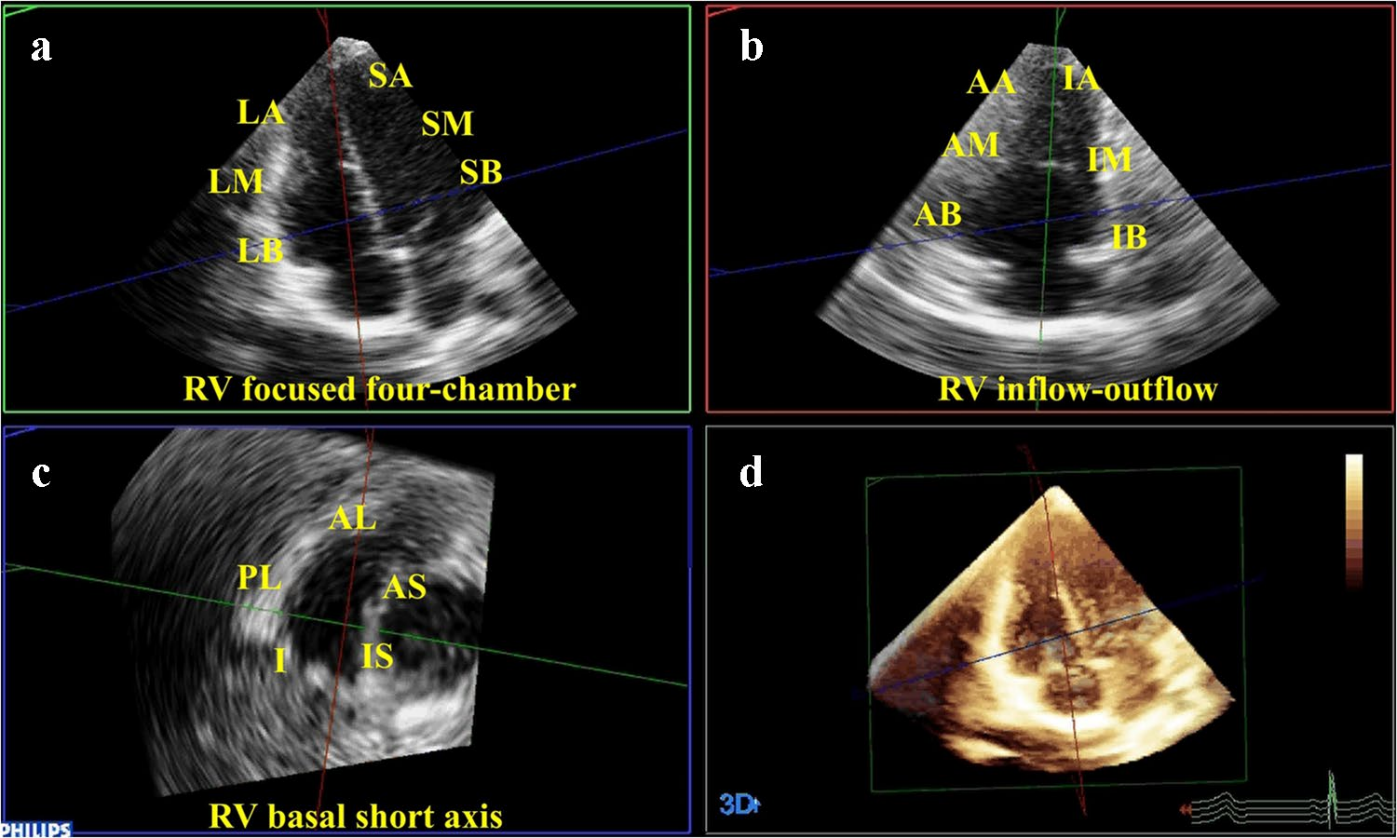
Using the three right ventricular (RV) views—RV-focused four-chamber view (**a**), RV inflow-outflow tract view (**b**), and basal short-axis view (**c**)—from a 6-year-old boy diagnosed with noncompaction cardiomyopathy as an example, this demonstrates the division of the RV wall into 17 segments. **d** The multiplanar reconstruction tool from the QLAB was used to extract the three views. *AA* anterior apical wall, *AB* anterior basal wall, *AL* anterolateral wall, *AM* anterior mid wall, *AS* anteroseptal wall,* I* inferior wall, *IA* inferior apical wall, *IB* inferior basal wall, *IM* inferior mid wall, *IS* inferoseptal wall, *LA* lateral apical wall, *LB* lateral basal wall, *LM* lateral mid wall, *PL* posterolateral wall, *SA* septal apical wall, *SB* septal basal wall, *SM* septal mid wall

Systematic evaluation of 3DE dataset image quality was performed by Q.L., a cardiovascular sonographer with 20 years of clinical experience. The RV basal short-axis, four-chamber, and inflow-outflow tract views were extracted using the multiplanar reconstruction tool in the 3-D Viewer software (QLAB, Philips Healthcare). Based on these views, each RV wall was divided into 17 segments (Fig. 3): lateral apical wall, lateral midwall, lateral basal wall, septal apical wall, septal midwall, septal basal wall, anterior apical wall, anterior midwall, anterior basal wall, inferior apical wall, inferior midwall, inferior basal wall, anterolateral wall, posterolateral wall, inferior wall, anteroseptal wall, inferoseptal wall [13]. Endocardial border visualization was scored as 0 (not visible), 1 (partially visible), or 2 (visible) for each segment. Overall image quality was classified by the sum of all segment scores: < 18=poor, 18 ~ 23=fair, and >23=good, based on established criteria [13].

### Cardiac magnetic resonance imaging acquisition and analysis

Cardiac MR images were obtained with a 1.5-T (T) scanner (MAGNETOM Area, Siemens Healthcare, Erlangen, Germany) equipped with an 18-channel phased-array cardiovascular coil. In each child, the long axis of the heart was identified using retrospective ECG-gated localization spin-echo sequences. Steady-state free precession dynamic gradient-echo cine loops were then acquired with retrospective ECG-gated and parallel imaging sensitivity encoding, and the RV short-axis view was acquired with the following parameters: 6-mm slice thickness of the imaging planes, 340 × 255 mm field of view, 256 × 205 scan matrix, 80° flip angle, 2.93/1.16 ms repetition/echo times, and 20 reconstructed cardiac phases.

**Fig. 4:**
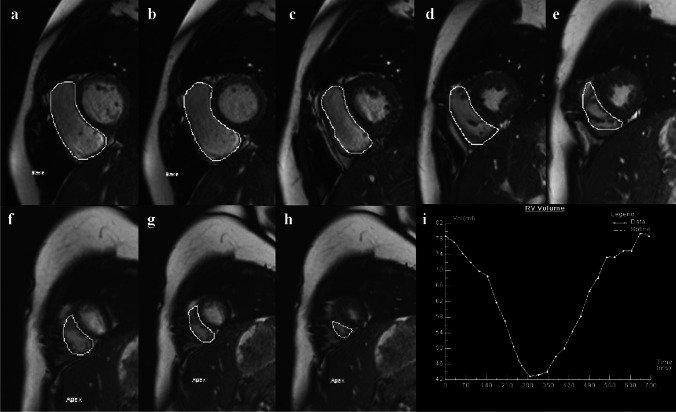
Partial right ventricular (RV) short-axis views obtained by cardiac magnetic resonance (MR) in a 10-year-old boy with patent foramen ovale, depicting RV basal segments (**a**,** b**), mid-cavity segments (**c**–**e**), and apical segments (**f**–**h**). **i** Volume-time curve of the RV generated by cardiac MR image analysis software

The RV images were analyzed offline using commercial software (Argus; Siemens Medical Solutions, Erlangen, Germany). Manual tracing of RV endocardial contours on all short-axis slices at end-diastolic and end-systolic frames was performed by Y.Y. (radiology, cardiac MR specialist, 7 years of experience), who was an investigator blinded to echocardiographic results. The endocardial trabeculae were included within the RV cavity. Finally, RVEDV, RVESV, and RVEF were automatically calculated by the software (Fig. 4).

### Reproducibility analysis

In this study, automated analysis was achieved without manual editing in 29 children, with repeated measurements consistently yielding identical values (100% reproducible). Intra- and inter-observer variability were evaluated in the remaining 53 children requiring manual editing and evaluated through the intra-class correlation coefficients (ICCs) and coefficients of variation (CoV). Intra-observer variability was assessed by the same investigator who repeated the measurements a month later, and inter-observer variabilities were determined by a second investigator.

### Statistical analysis

Continuous variables are presented as mean ± standard deviation (SD) or median and interquartile range. Categorical variables are reported as percentages. RV volumes and EF obtained by the fully automated 3DE were compared with the cardiac MR measurements using the Wilcoxon signed-rank test. The correlation and agreement between the two sets of measurements were assessed using Spearman’s correlation for non-normally distributed variables, Pearson correlation for normally distributed variables, and Bland–Altman analysis to calculate the bias and the limits of agreement (LOA).

Values of *P* <0.05 were considered significant. The statistical analyses were performed using SPSS version 26.0 (SPSS, Inc, Chicago, IL) and Prism 8.0.2 (GraphPad Software, San Diego, CA).

## Results

### Patient characteristics

Of the 90 consecutive children, eight were excluded due to arrhythmia (*n*=3), clinically unstable state, or failure to cooperate in completing the cardiac MR examination (*n*=3), receiving treatments that could impact the RV function between the time of cardiac MR and the time of 3DE (*n*=2). Consequently, a total of 82 participants (mean age, 11 ± 4 years; 51 boys [62%]) were ultimately included. Clinical characteristics of the cohort are summarized in Table 1. Among the 82 children, 14 patients were diagnosed with dilated cardiomyopathy, four with hypertrophic cardiomyopathy, five with restrictive cardiomyopathy, two with heart valve disease, six with myocarditis, eight with leukemia, and 41 with another broad-spectrum diagnosis.

**Table 1:** Clinical characteristics of the studied population

Population, *n*=82	Mean ± SD, or *n* (%)
Parameters	
Boys (%)	51 (62)
Age (years)	11 ± 4
Height (cm)	146 ± 25
Weight (kg)	41 ± 21
BSA (m^2^)	1.27 ± 0.43
HR (bpm)	83 ± 20
SBP (mmHg)	107 ± 14
DBP (mmHg)	68 ± 10
Diagnosis	
Dilated cardiomyopathy	14 (17.1)
Hypertrophic cardiomyopathy	4 (4.9)
Restrictive cardiomyopathy	5 (6.1)
Heart transplant	2 (2.4)
Heart valvular disease	2 (2.4)
Myocarditis	6 (7.3)
Leukemia	8 (9.8)
Others	41 (50.0)

*BSA* body surface area,* DBP* diastolic blood pressure, *HR* heart rate,* SBP* systolic blood pressure, *SD* standard deviation

### Comparison of right ventricular volumes and right ventricular ejection fraction between three-dimensional echocardiography and cardiac magnetic resonance

**Fig. 5:**
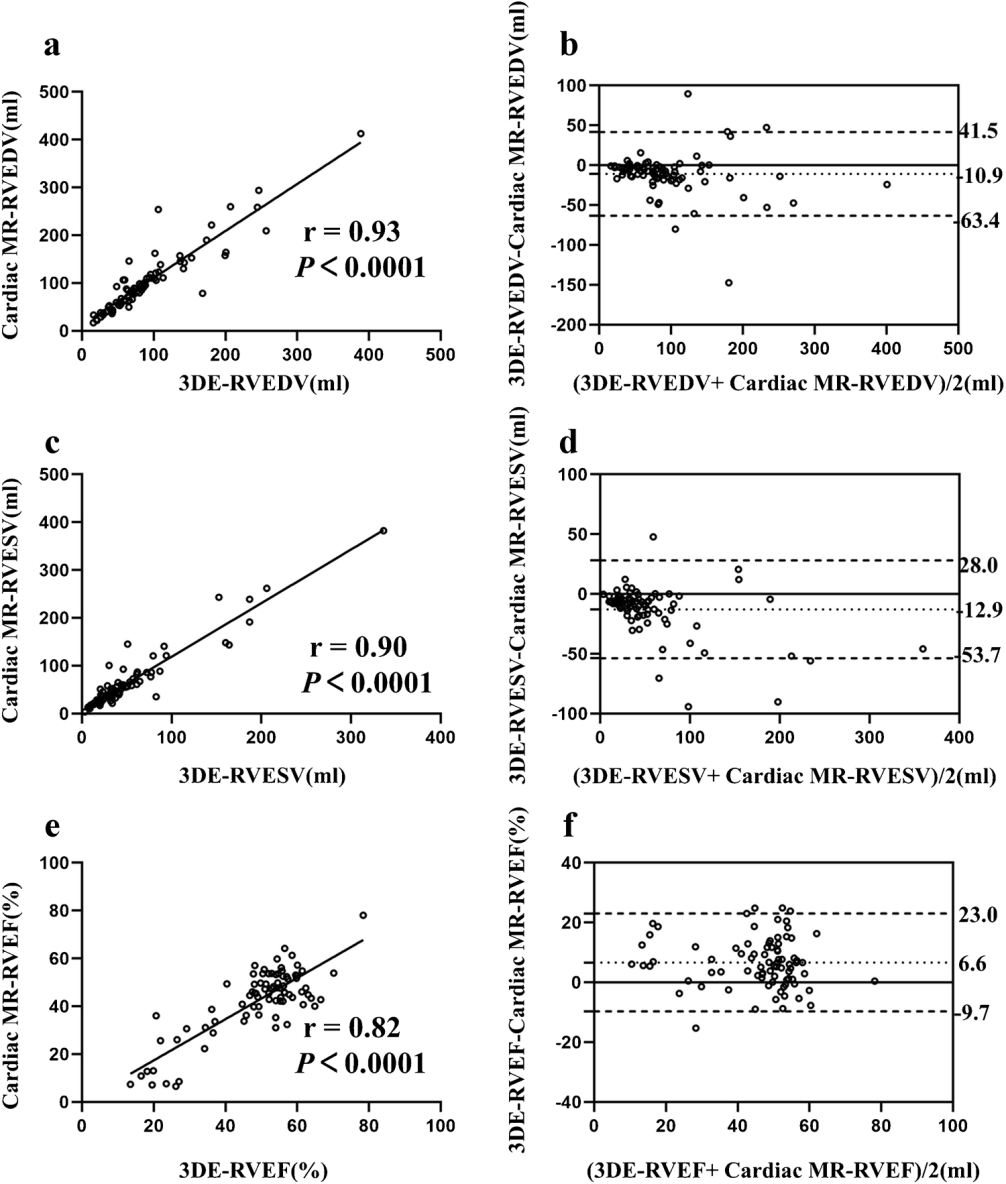
Correlation and Bland–Altman analysis for right ventricular (RV) volumes and ejection fraction (EF) between automated three-dimensional echocardiography (3DE) method and cardiac magnetic resonance (MR) measurements.** a**–**b** 3DE-RV end-diastolic volume (EDV) vs. cardiac MR-RVEDV. **c**–**d** 3DE-RV end-systolic volume (ESV) vs. cardiac MR-RVESV. **e**–**f** 3DE-RVEF vs. cardiac MR-RVEF

The study population encompassed a wide range of RV volumes and EF. The 3DE-RV quantification software analysis was feasible in all 82 patients (100%). As shown in Table 2 and Fig. 5, the fully automated 3DE slightly underestimated the RV volumes and overestimated the RVEF compared with cardiac MR (RVEDV: 71.4 [50.6, 103.0] ml vs. 85.1 [53.8, 119.0] ml, RVESV: 34.4 [20.9, 55.9] ml vs. 45.3 [26.6, 68.6] ml, RVEF: 48.9 ± 13.5% vs. 42.2 ± 14.2%; all *P* < 0.05). An excellent correlation of RV volumes and EF between the two methods was observed (*r*=0.93 for RVEDV, *r*=0.90 for RVESV, and *r*=0.82 for RVEF; all *P *<0.001).

**Table 2:** Comparison of right ventricular volumes and right ventricular ejection fraction by three-dimensional automated method against cardiac magnetic resonance measurements

Variable	Cardiac MR	Automated 3DE	Correlation	*P*-value	Bias	LOA
RVEDV (ml)	85.1 (53.8, 119.0)	71.4 (50.6, 103.0)	0.93	<0.001	−10.9	26.8 (−63.4, 41.5)
RVESV (ml)	45.3 (26.6, 68.6)	34.4 (20.9, 55.9)	0.90	<0.001	−12.9	20.8 (−53.7, 28.0)
RVEF (%)	42.2 ± 14.2	48.9 ± 13.5	0.82	<0.001	6.6	8.3 (−9.7, 23.0)

Data are expressed as mean ± standard deviation or median (interquartile range)

*3DE* three-dimensional echocardiography, *LOA* limits of agreement, *MR* magnetic resonance, *RVEDV* right ventricular end-diastolic volume, *RVEF* right ventricular ejection fraction, *RVESV* right ventricular end-systolic volume

### Effect of right ventricular function, and right ventricular size on the accuracyof fully automated three-dimensional echocardiography right ventricular quantification software

Table 3 presents the impact of RVEF subgroups on measurements from fully automated 3DE versus cardiac MR. The bias and LOA values for RV volumes and EF were larger in children with RVEF < 45% than in those with RVEF ≥ 45%. Table 4 demonstrates the influence of RV size on 3DE-derived RV volumes and EF compared to cardiac MR. The bias between 3DE and cardiac MR was also greater in children with RV dilatation than in those with normal RV size.

**Table 3:** The effects of right ventricular ejection fraction on the automated three-dimensional echocardiography measurements compared with cardiac magnetic resonance

Variable	Cardiac MR	Automated 3DE	Bias	LOA
Cardiac MR-derived-RVEF <45% group (*n*=40)				
RVEDV (ml)	94.2 (64.0, 161.2)	78.6 (55.6, 142.4)	−16.6	32.8 (−80.9, 47.7)
RVESV (ml)	58.9 (37.6, 120.9)	45.1 (23.6, 85.1)	−21.7	24.8 (−70.3, 26.9)
RVEF (%)	31.9 ± 12.8	42.4 ± 15.9	10.5	8.7 (−6.5, 27.5)
Cardiac MR-derived-RVEF ≥ 45% group (*n*=42)				
RVEDV (ml)	77.7 (48.7, 105.8)	67.7 (42.4, 96.1)	−5.5	18.2 (−41.1, 30.1)
RVESV (ml)	35.6 (23.4, 51.1)	30.3 (20.0, 42.2)	−4.4	11.2 (−26.3, 17.4)
RVEF (%)	52.1 ± 6.1	55.1 ± 6.4	2.9	6.1 (−9.0, 14.9)

Data are expressed as mean ± standard deviation or median (interquartile range)

*3DE* three-dimensional echocardiography, *LOA* limits of agreement, *MR* magnetic resonance, *RVEDV* right ventricular end-diastolic volume, *RVEF* right ventricular ejection fraction, *RVESV* right ventricular end-systolic volume

**Table 4:** The effects of right ventricular size on the automated three-dimensional echocardiography measurements compared with cardiac magnetic resonance

Variable	Cardiac MR	Automated 3DE	Bias	LOA
Normal RV size group (*n*=68)				
RVEDV (ml)	79.0 (50.0, 104.5)	68.0 (42.6, 88.3)	−7.3	17.5 (−41.7, 27.1)
RVESV (ml)	38.6 (24.1, 56.7)	31.7 (20.3, 45.1)	−7.9	12.7 (−32.8, 17.1)
RVEF (%)	46.9 ± 8.9	52.9 ± 9.2	6.0	8.5 (−10.7, 22.8)
RV dilation group (*n*=14)				
RVEDV (ml)	192.9 (110.5, 258.9)	190.2 (86.0, 245.0)	−28.4	49.8 (−126.0, 69.2)
RVESV (ml)	144.4 (91.2, 240.0)	122.2 (49.7, 187.0)	−37.1	33.3 (−102.4, 28.1)
RVEF (%)	19.8 ± 14.0	29.2 ± 14.1	9.5	6.8 (−3.9, 22.9)

Data are expressed as mean ± standard deviation or median (interquartile range)

*3DE* three-dimensional echocardiography, *LOA* limits of agreement, *MR* magnetic resonance, *RV* right ventricular, *RVEDV* right ventricular end-diastolic volume, *RVEF* right ventricular ejection fraction, *RVESV* right ventricular end-systolic volume

### Image quality of three-dimensional echocardiography analysis

Supplementary Material 1 shows the details of the 3DE image quality results for each segment, and the distribution of the image quality in the study group is summarized in Table 5. The fully automated 3DE analysis was accurate in 29 children (35%) without any manual editing and required only 8 ± 2 s. In the remaining 53 children (65%), manual editing was needed, prolonging the analysis time to 114 ± 46 s. Only a small percentage of children with poor (10%) and fair (23%) image quality could be analyzed without manual editing, in contrast to the subgroup of children with good image quality in whom fully automated 3DE analysis achieved 48% feasibility.

**Table 5:** Distribution of the image quality and the accuracy of the automated analysis results

Dataset quality	Patients (%)	Quality score (SD)	Accurate automated analysis (%)
Total	82	24.0 ± 5.2	29 (35.4)
Image quality subgroups			
Poor, <18	10 (12.2)	14.4 ± 1.9	1 (10.0)
Fair, 18–23	26 (31.7)	20.8 ± 1.8	6 (23.1)
Good, >23	46 (56.1)	27.4 ± 2.6	22 (47.8)

Data are expressed as mean ± standard deviation (SD) or* n* (%)

*SD* standard deviation

Among the three subgroups stratified by good, fair, and poor image quality, results similar to those above showed that the fully automated 3DE slightly underestimated the RV volumes and overestimated the RVEF compared to cardiac MR measurements (Table 6). The RV volumes and EF determined by 3DE in the good and fair image quality subgroup had higher correlations with the cardiac MR values than those in the poor image quality subgroup (Table 6).

**Table 6:** The effects of image quality on the automated three-dimensional echocardiography measurements compared with cardiac magnetic resonance

Variable	Cardiac MR	Automated 3DE	Correlation	*P*-value	Bias	LOA
Image quality good (*n*=46)						
RVEDV (ml)	79.5 (44.7, 127.0)	71.4 (41.9, 106.8)	0.97	<0.001	−9.1	26.7 (−61.4, 43.3)
RVESV (ml)	41.2 (22.7, 69.6)	35.6 (19.8, 57.1)	0.96	<0.001	−11.0	19.3 (−48.8, 26.8)
RVEF (%)	42.0 ± 15.2	47.8 ± 15.5	0.87	<0.001	5.8	7.9 (−9.7, 21.3)
Image quality fair (*n*=26)						
RVEDV (ml)	92.9 (62.5, 119.0)	71.7 (53.0, 112.5)	0.88	<0.001	−10.9	28.7 (−67.2, 45.4)
RVESV (ml)	50.4 (34.8, 67.3)	33.3 (21.0, 65.4)	0.83	<0.001	−14.9	24.0 (−61.8, 32.1)
RVEF (%)	41.3 ± 14.0	49.6 ± 11.8	0.76	<0.001	8.3	9.2 (−9.7, 26.4)
Image quality poor (*n*=10)						
RVEDV (ml)	95.2 ± 36.2	75.6 ± 29.0	0.79	0.006	−19.6	22.0 (−62.8, 23.6)
RVESV (ml)	52.6 ± 25.7	35.7 ± 14.8	0.60	0.068	−16.9	20.6 (−57.3, 23.6)
RVEF (%)	45.9 ± 9.6	52.6 ± 7.6	0.43	0.210	6.8	9.4 (−11.6, 25.1)

Data are expressed as mean ± standard deviation or median (interquartile range)

*3DE* three-dimensional echocardiography, *LOA* limits of agreement, *MR* magnetic resonance, *RVEDV* right ventricular end-diastolic volume, *RVEF* right ventricular ejection fraction, *RVESV* right ventricular end-systolic volume

### Reproducibility of three-dimensional echocardiography measurements

Supplementary Material 2 demonstrates excellent intra- and inter-observer reproducibility for all 3DE parameters, with CoV <15% and ICC >0.85.

## Discussion

Despite previous studies having investigated the feasibility and accuracy of 3DE in assessing pediatric RV volume and function in children with various pathologies using cardiac MR as the gold standard, the novelty of this study is derived from the inclusion of a large sample of children with normal heart anatomy and strict control of the interval between echocardiography and cardiac MR examinations within 24 h. The main findings of our study are as follows: (1) The novel 3DE-RV quantification software was feasible in all 82 children (100%), and measurements of RV volumes and function obtained by the novel software showed good agreement with cardiac MR values; (2) greater accuracy was achieved in children with RVEF ≥ 45%, normal RV size, and good 3DE image quality; (3) the accuracy of the fully automated 3DE was impacted by image quality; (4) the fully automated 3DE-RV methods yielded accurate measurements in 35% of children without any adjustment, enabling rapid analysis with excellent reproducibility. The combination of rapid analysis, excellent reproducibility, and accuracy supports the routine adoption of this method in clinical settings.

Cardiac MR has traditionally been used to assess RV volumes and EF due to its precision, consistency, and independence from geometric assumptions. Based on American Society of Echocardiography/European Association of Cardiovascular Imaging chamber quantifications guidelines [15], 3DE-derived RV volume and EF are currently recommended as the standard for assessing RV size and function. 3DE overcomes specific limitations of cardiac MR, including claustrophobia and challenges with intermittent breath-holding. Reproducibility of 3DE-derived RV volumetric measurements has been established in children with normal and pathological hearts [19, 20]. However, current semi-automated 3DE-RV quantification software requires a tedious analysis process and specialized training, limiting its clinical adoption. To address these constraints, a novel fully automated 3DE-RV quantification software employing machine learning algorithms has been developed. Accurate assessment of RV volumes and EF in adults using this automated software was demonstrated by Genovese et al. [13]. Nevertheless, the accuracy of pediatric RV function quantification with this software remains unverified.

Achieving rapid and accurate measurement of RV function is crucial for the diagnosis and prognostic assessment of various cardiac diseases in children. This pediatric population presents unique challenges, as long-term examination cooperation is difficult to achieve, and their relatively fast heart rate and poor respiratory coordination may theoretically pose additional challenges to fully automated 3DE software. Our results revealed the accuracy of the new fully automated 3DE-RV quantification software in a pediatric population with diverse RV sizes and functions, using the cardiac MR as the gold standard. It was found that 3DE-derived RV volumes were lower than cardiac MR assessments, with low percentage discrepancy for EDV measurements and higher discrepancy for ESV in the entire cohort, consistent with previous studies [9, 20]. A key factor contributing to differences in RV volume measurements between cardiac MR and echocardiography is the variation in endocardial border delineation [21, 22]. Additionally, previous research indicates that RVEF evaluation shows greater variability between 3DE and cardiac MR than RV volume measurements [10]. The lower RVEF correlation reported by Muraru et al. [23] may be explained by semi-automated detection. After manual refinement was performed, the correlation between 3DE-derived RVEF and cardiac MR-derived RVEF improved [23]. In our study, manual editing was necessary in 65% of patients, potentially affecting RVEF measurement accuracy.

Additionally, the fully automated 3DE-RV approach was characterized by rapid analysis and excellent reproducibility in this study. Consistent results are produced for identical images by the automated method, regardless of investigator, as the analysis is performed using identical machine learning algorithms. This consistency is essential for precise longitudinal assessment of pediatric RV function during follow-up, with potential clinical implications.

A greater underestimation of RV volumes by 3DE was observed in children with dilated right ventricles compared to those without, which is consistent with previous studies of 3DE-RV quantification software [11, 19, 20]. Typically, patients with RV dilatation have abundant trabeculae, making accurate tracking of the endocardial border more challenging for fully automated software. This could be a reason for varied bias ranges in 3DE measurements. Therefore, trabeculae and papillary muscles should be included in the RV cavity during endocardial border tracking. Of note, 14 children (17%) in this study had RV dilatation. In these cases, abnormal RV morphology and incomplete 3-D full-volume image acquisition may occur, potentially leading to inaccurate endocardial identification and consequent greater 3DE volume underestimation. Besides, lower accuracy was noted in children with reduced RVEF using the fully automated 3DE-RV quantification software. As discussed in other studies [24–26], these children often exhibit RV dilation with abundant trabeculae, posing greater challenges for RV volume and function measurement.

Considering the retrosternal location of the right ventricle and its complex crescent shape, obtaining high-quality 3DE-RV images can be challenging. In our study, the image quality scores indicated incomplete visualization of the basal and middle segments of the RV anterior wall, due to their distance from the probe and obstruction by the sternum.

The success rate of the fully automated approach was dependent on image quality. In the present study, the fully automated analysis success rate in the good image quality group was 48%, lower than the 70% reported in a previous adult study with good image quality [13]. A possible explanation is that the machine learning algorithm of this software is based on adult 3DE image data; consequently, it may fail to correctly identify pediatric RV anatomy. Additionally, the relatively limited sample size (*n*=56) in the prior study versus the larger unselected consecutive cohort in our study may contribute to the discrepancy. Nevertheless, the overall automated analysis success rate was similar between studies (35% vs. 32%) [13]. The automated 3DE-RV quantification software is considered a valuable tool for pediatric RV assessment. Through the refinement of software algorithms and the accumulation of larger pediatric datasets, the success rate and accuracy of fully automated 3DE are expected to improve.

Several limitations should be acknowledged in this study. First, this was a single-center study. Acquiring 3DE images in children is more challenging than in adults, and the sample size and disease spectrum were limited. Therefore, multicenter studies with expanded sample sizes should be conducted to further assess the accuracy of fully automated 3DE for quantifying RV volume and function. Second, suboptimal cooperation in some young children required sedation for echocardiography and cardiac MR examinations. Although sedation improves procedural feasibility by minimizing respiratory motion artifacts, maintaining stable positioning, and reducing image acquisition time [27], it introduces potential confounders [27–29]: (1) Altered respiratory patterns (e.g., reduced rate or increased depth) may exacerbate respiratory motion artifacts, impairing image clarity; (2) heart rate variability (including bradycardia or arrhythmias) can lead to inconsistent cardiac cycle capture; (3) individual variations in sedative response may amplify physiological changes. Notably, the benefits of sedation generally outweigh its risks in pediatric echocardiography. Future studies could compare pre- vs. post-sedation echocardiographic image quality to determine sedation’s effect on fully automated 3DE-RV quantification accuracy. Third, unified standards for 3DE assessment of pediatric RV volume and function are lacking in Asian children. Lastly, this study was performed using the existing X5-1 probe, since the X7 3D probe was unavailable at our institution. Although the X5-1 probe has lower resolution for pediatric imaging, potentially affecting image quality, its stable performance and suitability for our research objectives were confirmed through extensive preliminary testing [30].

## Conclusions

Fully automated 3DE-RV methods achieved accurate results in 35% of children without any adjustment, providing rapid analysis and excellent reproducibility. The RV volumes and EF measured by the fully automated 3DE method correlated well with those determined using the gold standard cardiac MR. Furthermore, measurements were more accurate in children with RVEF ≥ 45%, normal RV size, and good 3DE image quality. Therefore, the novel 3DE software may offer a valid tool for rapid and accurate assessment of RV function in children with normal heart anatomy.

## Supplementary Information

Below is the link to the electronic supplementary material.

ESM 1 DOCX (18.5 KB)

ESM 2 DOCX (16.9 KB)

## Data Availability

The datasets analysed during the current study are available from the corresponding author on reasonable request.
