# Supplementary material for: Feasibility and accuracy of the fully automated three-dimensional echocardiography right ventricular quantification software in children: validation against cardiac magnetic resonance
Source: Pediatr Radiol. 2025 Jul 18;55(12):2566–78. doi: 10.1007/s00247-025-06330-2 (PMC12602562; doi:10.1007/s00247-025-06330-2)
Supplement: Supplementary file 1 — DOCX (18.5 KB) [file 247_2025_6330_MOESM1_ESM.docx]

**Supplemental Table 1** Percentage of image quality scores for each right ventricular segment in the 82 children

| RV Views and Endocardial Segments | Not Visible | Partially Visible | Visible |
| --- | --- | --- | --- |
| Short axis |  |  |  |
| Basal |  |  |  |
| Anteroseptal | 4 | 33 | 63 |
| Inferoseptal | 1 | 15 | 84 |
| Inferior | 4 | 22 | 74 |
| Posterolateral | 28 | 43 | 29 |
| Anterolateral | 41 | 51 | 7 |
| Four-chamber |  |  |  |
| Septum |  |  |  |
| Basal | 0 | 5 | 95 |
| Mid | 0 | 1 | 99 |
| Apex | 2 | 39 | 59 |
| Lateral |  |  |  |
| Basal | 2 | 15 | 83 |
| Mid | 7 | 32 | 61 |
| Apex | 15 | 61 | 24 |
| Inflow-outflow |  |  |  |
| Inferior |  |  |  |
| Basal | 0 | 13 | 87 |
| Mid | 0 | 11 | 89 |
| Apex | 10 | 62 | 28 |
| Anterior |  |  |  |
| Basal | 45 | 29 | 26 |
| Mid | 44 | 34 | 22 |
| Apex | 33 | 60 | 7 |

Data are expressed as percentages (%)

*RV* right ventricular
