# Supplementary material for: Feasibility and accuracy of the fully automated three-dimensional echocardiography right ventricular quantification software in children: validation against cardiac magnetic resonance
Source: Pediatr Radiol. 2025 Jul 18;55(12):2566–78. doi: 10.1007/s00247-025-06330-2 (PMC12602562; doi:10.1007/s00247-025-06330-2)
Supplement: Supplementary file 2 — DOCX (16.9 KB) [file 247_2025_6330_MOESM2_ESM.docx]

**Supplemental Table 2** Reproducibility of the automated three-dimensional echocardiography method

| Variable | ICC (95%CI) | CV (%) |
| --- | --- | --- |
| Intra-observers (*n*=53) | |  |
| RVEDV (ml) | 0.992(0.986-0.995) | 6.2 |
| RVESV (ml) | 0.992(0.986-0.995) | 11.1 |
| RVEF (%) | 0.908(0.845-0.946) | 9.6 |
| Inter-observers (*n*=53) | |  |
| RVEDV (ml) | 0.970(0.949-0.983) | 7.7 |
| RVESV (ml) | 0.974(0.956-0.985) | 14.4 |
| RVEF (%) | 0.898(0.830-0.940) | 9.6 |

*CI* Confidence Interval*, CV* Coefficient of Variation, *ICC* intra-class correlation coefficient, *RVEDV* right ventricular end-diastolic volume, *RVEF* right ventricular ejection fraction, *RVESV* right ventricular end-systolic volume
